# microRNA-23a in Human Cancer: Its Roles, Mechanisms and Therapeutic Relevance

**DOI:** 10.3390/cancers11010007

**Published:** 2018-12-20

**Authors:** Ning Wang, Hor-Yue Tan, Yi-Gang Feng, Cheng Zhang, Feiyu Chen, Yibin Feng

**Affiliations:** 1 School of Chinese Medicine, Li Ka Shing Faculty of Medicine, The University of Hong Kong, 10 Sassoon Road, Pokfulam, Hong Kong, China; ckwang@hku.hk (N.W.); hyhtan@hku.hk (H.-Y.T.); zttc@hku.hk (C.Z.); fychen@connect.hku.hk (F.C.); 2 Guanghua School of Stomatology, Sun Yat-Sen University, Guangzhou 510006, China; ygfeng18@hotmail.com

**Keywords:** miR-23a, cancer diagnosis, cancer prognosis, cancer progression, cancer drug sensitivity, tumor microenvironment

## Abstract

microRNA-23a (miR-23a) is one of the most extensively studied miRNAs in different types of human cancer, and plays various roles in the initiation, progression, and treatment of tumors. Here, we comprehensively summarize and discuss the recent findings about the role of miR-23a in cancer. The differential expression of tissue miR-23a was reported, potentially indicating cancer stages, angiogenesis, and metastasis. miR-23a in human biofluid, such as plasma and salivary fluid, may be a sensitive and specific marker for early diagnosis of cancer. Tissue and circulating miR-23a serves as a prognostic factor for cancer patient survival, as well as a predictive factor for response to anti-tumor treatment. The direct and indirect regulation of miR-23a on multiple gene expression and signaling transduction mediates carcinogenesis, tumor proliferation, survival, cell migration and invasion, as well as the response to anti-tumor treatment. Tumor cell-derived miR-23a regulates the microenvironment of human cancer through manipulating both immune function and tumor vascular development. Several transcriptional and epigenetic factors may contribute to the dysregulation of miR-23a in cancer. This evidence highlights the essential role of miR-23a in the application of cancer diagnosis, prognosis, and treatment.

## 1. Introduction

Evidence on the role of microRNAs (miRNAs) in human cancer has been accumulating since the first observation by Croce et al., of miRNAs dysregulation in cancer [1]. The deregulated miRNA expression, which may affect up to 50 mRNA encoding proteins [2], is mainly associated with the oncogenic switch of intracellular signaling in cancer. The idea that “aberrant miRNA expression is the rule rather than the exception in cancer” has been supported by a large volume of data obtained, in recent studies [3]. These suggest that miRNAs can control carcinogenesis, cancer cell proliferation and survival, metastasis, as well as cellular response to anti-cancer treatment [4]. As the profiling of miRNAs dramatically distinguishes between the healthy population and cancer patients, tissue and biofluid miRNAs might be applied for diagnostic and prognostic purposes.

Among the cancer-associated miRNAs, microRNA-23a (miR-23a) was the recent focus of study. The human miR-23a is located at chromosome 19 of the human genome and was transcribed as a part of miR-23a–27a–24-2 cluster [5]. Dysregulation of miR-23a has been reported in various human diseases, such as ischemia-reperfusion injury, coronary heart diseases, and cancer [6]. In 2012, as one of the earliest laboratories to study the role and function of miR-23a in human cancer, we identified miR-23a as one of the key molecules that regulate the sensitivity of liver cancer cells to topoisomerase II inhibitor treatment, in which topoisomerase I was the direct target of miR-23a [7]. The role of miR-23a in human cancer has been explored by numerous clinical, translational, and basic studies. The increasing amount of scientific evidence has confirmed the therapeutic relevance of miR-23a in human cancer, for which a critical review is necessary. In this review, we focus on the role of miR-23a in human cancer, which has not yet been critically reviewed elsewhere, in order to determine its therapeutic potential, based on the clinical and biological insight into its function in cancer.

## 2. Diagnostic and Prognostic Values of miR-23a in Cancer

### 2.1. miR-23a Expression in Cancer

The expression pattern of miR-23a has been extensively studied and compared in the tumor and non-tumor tissues of human cancer (Table 1). In most cancer types, the expression of miR-23a is dysregulated, although up-regulation or down-regulation has been differentially observed, subject to the particular cancer type. miR-23a is often down-regulated in both acute and chronic myelogenous leukemia [8,9,10], with the only exception reported in a rare form of myeloid leukemia, named acute erythroid leukemia, where the expression of miR-23a is up-regulated [11]. The expression pattern of miR-23a in lymphocytic leukemia has not been reported. In solid tumors, miR-23a is down-regulated in most of the cancer occurring in the genitourinary system, such as prostate carcinoma [12,13], endometrial endometrioid adenocarcinoma [14], and nephroblastoma [15]. Although the exact reason why miR-23a expression in cancer of the reproductive and urinary system is down-regulated remains unclear, it is postulated that the similar pattern of miR-23a is due to their shared embryological origin from the intermediate mesoderm. This could be supported by the fact that expression of miR-23a in osteosarcoma, carcinoma of the intermediate mesoderm-derived bone system, was also down-regulated [16,17]. In contrast, carcinomas originating from the head and neck, respiratory and digestive systems, often demonstrate an over-expression of miR-23a.

### 2.2. Clinical Significance of Tissue Expressing miR-23a in Cancer

Several clinical evidences have supported the idea that dysregulation of miR-23a, as stated above, is correlated with the clinical features of human cancer. miR-23a expression was suggested to indicate a later clinical stage, as well as the extent of metastasis. In laryngeal cancer, a high expression of miR-23a in tumor tissue was significantly correlated with more advanced clinical stage and lymph node metastasis (*p* < 0.05 and *p* < 0.01, respectively) [40]. Zhu et al. found that, in the esophageal squamous cell cancer (ESCC) tissue, miR-23a expression was related to tumor differentiation (*p* < 0.05) [24]. In hepatocellular carcinoma (HCC), miR-23a was significantly associated with TNM stage and tumor size (*p* = 0.041 and 0.047, respectively) [37]. Tang et al. showed that, in colon carcinoma, the expression of miR-23a is positively associated with clinical stages (*p* = 0.029) and depth of invasion (*p* = 0.000) [49]. miR-23a expression in non-small cell lung cancer (NSCLC) tissues was correlated with smoking habit (*p* = 0.001), tumor size (*p* = 0.002), TNM stage (*p* = 0.001), lymph node metastasis (*p* < 0.001), and tumor differentiation (*p* = 0.004) [42]. A similar observation was also reported in studies of several other kinds of cancer. Bao et al. found that miR-23a expression was up-regulated at the metastatic and pre-metastatic stages of nasopharyngeal carcinoma (NPC), with an increased level of microvessel density, in the tumor tissue [50]. Expression of miR-23a was positively correlated with the tumor differentiation degree, lymph node metastasis, and clinical stages in ovarian cancer [51]. Ma et al. reported that miR-23a expression was significantly higher in the breast tumor tissues of patients with lymph node metastasis [52]. This evidence suggests the significance of miR-23a, in terms of its correlation with the staging, differentiation, and metastasis of cancer.

### 2.3. miR-23a as a Non-Invasive Marker in Cancer Diagnosis

miR-23a was identified as over-expressed in the serum of various types of human cancer, including breast [53,54], gastric [55], pancreatic [56], and esophageal squamous cell carcinoma [57], as well as in malignant astrocytoma [58]. Circulating miR-23a is, in contrast, down-regulated in patients with benign tumors of the salivary gland [59] and metastatic melanoma [18]. The expression of miR-23a in plasma, alone or in combination with a panel of other miRNAs, may be correlated with a specific clinical outcome of cancer patients, indicating the potential of miR-23a as a non-invasive marker in cancer diagnosis. For instance, a more aggressive phenotype, demonstrating more microscopic tumor infiltration at the resection margin and more perineural invasion, was found in pancreatic tumor tissues expressing high levels of miRNAs, including miR-21, miR-23a, and miR-27a [56]; miR-23a over-expression is associated with the tumor differentiation degree, lymph node metastasis, and tumor invasion [47]. In addition to its detection in the plasma of pancreatic cancer patients, Humeau et al. found that miR-23a was over-expressed in the saliva of pancreatic cancer patients with precursor lesions [60]. Khare et al. identified a panel of repressed plasma miRNAs, including miR-23a, and suggested that it may be a helpful diagnostic marker for differentiation of B-cell lymphoma and Hodgkin lymphoma [61].

Yong et al. found that miR-23a is significantly up-regulated in the serum of patients with colon cancer. Combined with miR-193a-3p and miR-388-5p, miR-23a yields a receiver operating characteristic (ROC) curve area of 0.887 (80.0% sensitivity, 84.4% specificity, and 83.3% accuracy), demonstrating its ability as a classifier for colorectal cancer detection [62]. Further analysis showed that miR-23a is encapsulated in the exosome, and circulating exosomal miR-23a is up-regulated in the serum of early-stage colorectal cancer patients. Significant down-regulation of exosomal miR-23a was found after tumor resection, indicating the potential of exosomal miR-23a for colon cancer detection [63]. This was supported by another study that showed that miR-23a expression is significantly down-regulated in serum EpCAM+ extracellular vesicles of colon cancer patients, after surgery [64]. However, in a study by Vychytilova-Faltejskova et al., miR-23a expression was found to be down-regulated in the serum of colorectal cancer patients, and a combination of serum miRNAs consisting of miR-23a, miR-27a-3p, miR-142-5p, and miR-376c-ep was proposed to be used for diagnosis of early-stage (T1-4N0M0, 0.877) colorectal cancer (0.917, 89% sensitivity and 81% specificity) [65].

### 2.4. The Prognostic Value of miR-23a in Cancer

Regarding the role of miR-23a as a prognostic factor in human cancer, the differential expression of miR-23a in various tumors may indicate a differential association with patient survival. Li et al. showed that the up-regulated tissue miR-23a predicted cancer progression and poor prognosis in patients [31]. In HCC patients, high miR-23a expression in cancer tissues is a risk factor for overall and recurrence-free survival [37]. In large B-cell lymphoma, miR-23a expression is associated with poor overall patients’ survival (*p* = 0.029); however, no significant association between the expression of miR-23a and other clinical features, especially the Ann Arbor stage, was observed [38]. Over-expression of miR-23a predicted poor overall survival in pancreatic cancer [47], as well as shorter survival after surgical resection (hazard ratio (HR) 3.21, 95% confidence interval (CI) 1.78–5.78) [56]. Similar observation on the association of miR-23a over-expression with poor patients’ survival was also observed in other cancers, including Ewing’s sarcoma [55], laryngeal cancer [40], malignant pleural mesothelioma (MPM) [43], NSCLC [42], and ovarian cancer [51]. In contrast, a low level of miR-23a expression in the prostate tumor tissue predicts a poor patient prognosis [13]. A meta-analysis including different kinds of human cancer suggested that an overall high level of miR-23a predicted the overall patients’ survival (HR = 2.33, 95% CI: 1.18–4.58; *p* = 0.014) but not a disease-free/recurrence-free survival (HR = 1.13, 95% CI: 0.37–3.44; *p* = 0.836). In a subgroup analysis, miR-23a expression predicted poor overall survival in digestive system cancer but not in respiratory cancer [66].

**Figure 1:**
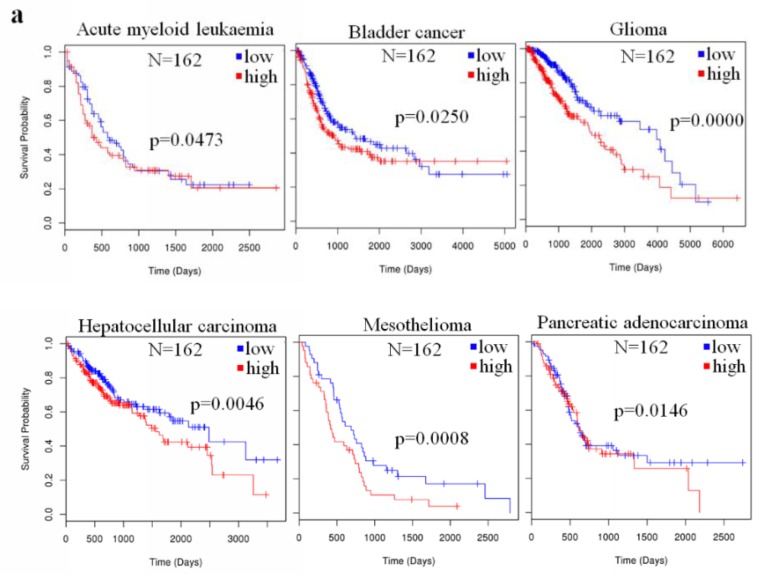

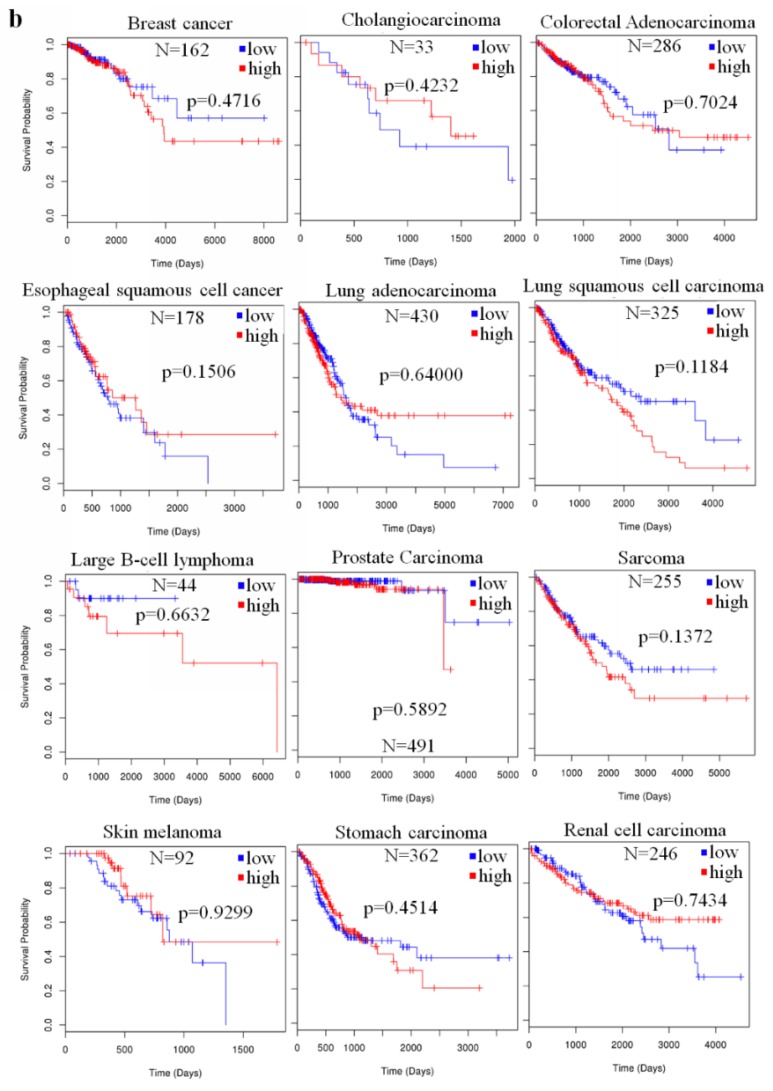
The prognostic value of microRNA-23a (miR-23a) in human cancer. The Cancer Genome Atlas (TCGA) data was retrieved using the Linkedomics platform, and patients were grouped on the basis of the expression of the miR-23a in tumors [67]. (**a**) Shows six cancer types, in which overall survival of patients is significantly correlated with the miR-23a expression; (**b**) shows other cancer types in which overall survival of patients is not significantly correlated with the miR-23a expression though miR-23a is significantly de-regulated in these tumors.

To analyze the prognostic value of miR-23a in the survival of patients with different kinds of cancer on the same platform, we retrieved miR-23a expression and patients information from The Cancer Genome Atlas (TCGA) database, using the Linkedomics platform [67]. In each type of cancer, patients were divided into two groups, based on tumor miR-23a expression, either lower or higher than the median level. The survival of patients with the six types of cancer, including acute myeloid leukemia (AML), bladder cancer, glioma, HCC, MPM, and pancreatic adenocarcinoma were correlated with miR-23a expression level (Figure 1a), and a higher level of miR-23a, in these cancers, predicted a poorer overall survival. The miR-23a expression that was reported to be differentially expressed in other types of tumor tissues, as stated in Table 1, did not show any correlation with the patients’ overall survival (Figure 1b). Although these data may fluctuate with the sample size, as well as the reliability of the sequencing method in the miR-23a quantification, our summary suggests that miR-23a expression only exhibits a prognostic value in several particular types of human cancer.

Chemoresistance and radioresistance are main risk factors of poor prognosis in cancer patients undertaking chemo- and radio-therapy; previous studies have also focused on using miR-23a to predict acquired chemoresistance in cancer patients. Komatsu et al. used plasma miR-23a to predict the chemoresistance in ESCC patients receiving preoperative chemotherapy. A higher expression of miR-23a was observed in the plasma of patients with a lower histopathologic response than in those with a high histopathologic response or fewer residual tumor lesions (*p* = 0.0114) [57]. El-Halawany et al. found that a panel of the miRNAs, including miR-23a, may identify the response of the HCC patients to transarterial chemoembolization (TACE). miR-23a was significantly up-regulated in the non-responder group of the HCC patients [68]. miR-23a was found to be repressed in NPC with radioresistance, and a low expression of miR-23a predicted a poor patient survival [69].

## 3. Biological Role of the miR-23a in Human Cancer

### 3.1. Carcinogenesis

As an early epigenetic factor that senses the genomic response to environmental changes during carcinogenesis, miRNAs, including miR-23a, were widely reported to be dysregulated during malignant transformation of normal human tissues [70]. The involvement of miR-23a as a regulator of the change of robust gene expression, suggested its oncogenic role in the initiation of human cancer. The areca nut-chewing habit may lead to oral carcinogenesis. A study by Tsai et al. showed that cells treated with areca nut extract had a higher miR-23a expression. The over-expression of miR-23a is correlated with the DNA double-strand break repair and DNA damage, suggesting the role of miR-23a in areca nut-induced carcinogenesis [71]. Wang et al. studied the change in miRNA profiles during hepatocarcinogenesis and found that miR-23a expression was up-regulated through the activation of IL6/Stat3 signaling in the liver. The over-expression of the miR-23a directly targets Glucose-6-phosphatase (G6PC) and peroxisome proliferator-activated receptor gamma coactivator 1-alpha (PGC-1α), in the gluconeogenesis pathway, to reduce the protein expression, which facilitated carcinogenesis in the liver [72].

### 3.2. Cancer Cell Proliferation and Survival

**Figure 2:**
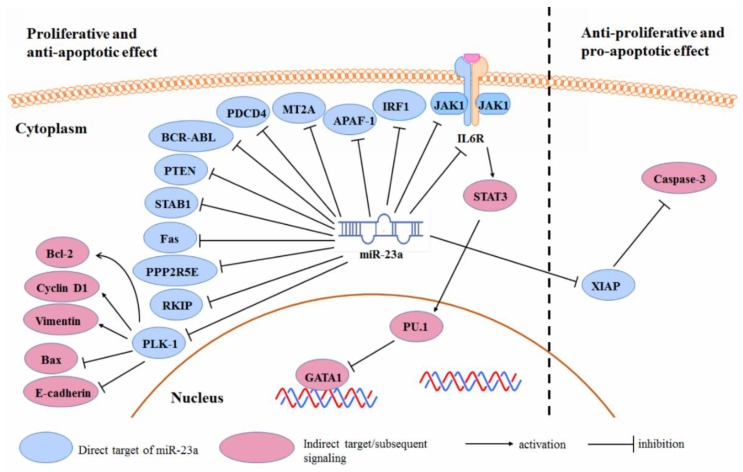
Regulation of the miR-23a on cancer cell proliferation and apoptosis.

Both positive and negative roles of the miR-23a, in cancer cell maintenance, were found in different kinds of cancer. As a robust cellular regulator of gene expression, miR-23a may target a broad range of mRNAs in cancer cells, by direct binding to their three prime untranslated region (3′-UTR), which in turn suppresses gene expression. The cell fate is the overall and summed results of both pro-cell death and anti-cell death signaling induced by the miR-23a-targeted genes [73]. In cancer cells, protein expression pattern might be totally different from the normal cells, due to the gene mutations that can lead to either expression amplification or loss, and different cancers may have different mutations. In this case, though the miR-23a is theoretically considered to regulate the same pool of genes, miR-23a may miss its match with some mutated gene. This could largely affect the related pro-/anti-cell death signaling and, thereby, the cell fate decision. In this case, when the expression of some target protein of the miR-23a is defective, in particular types of human cancer, the cell fate decided by the miR-23a expression could be in the opposite direction. The overall regulatory scheme of the miR-23a in determining cancer cell fate, is shown in Figure 2.

In acute erythroid leukemia (AEL) cells, over-expression of the miR-23a may induce apoptosis and erythropoiesis. Su et al. also identified that Janus kinase 1 (JAK1) is the direct target of the miR-23a in AEL cells [11]. The suppression of JAK1-inactivated Stat3-mediated globin transcription factor 1 (GATA1) activity through PU.1 improved the erythroid differentiation. Inhibiting miR-23a led to an oncogenic phenotype by activating the gp130/JAK1/Stat3 pathway, and a JAK1 inhibitor—ruxolitinib—helped with this oncogenic change [11]. Aghaee-Bakhtiari et al. suggested that IL6R inhibition by miR-23a in prostate cancer may inactivate the MAPK and JAK/STAT pathway, which contributes to a reduced cell proliferation and neoplastic transformation [12]. Forced expression of miR-23a in chronic myeloid leukemia leads to cellular senescence. This was dependent on the suppression of breakpoint cluster region protein/Abelson murine leukemia viral oncogene homolog 1 (BCR/ABL), whose 3’UTR was the direct target of the miR-23a [10]. The over-expression of miR-23a in pancreatic cancer cells, suppressed cell proliferation and promoted cell apoptosis. The polo-like kinase 1 (PLK-1) was identified as the direct target of the miR-23a in PC cells. miR-23a-induced PLK-1 expression led to the decreased expression of Bcl-2, Cyclin B1, vimentin, and an increased expression of Bax and E-cadherin [74].

The up-regulation of miR-23a in gastric cancer promoted cell proliferation and inhibited apoptosis [28]. This could be related to the reduced expression of IL6R by miR-23a; Zhu et al. suggested that miR-23a can target the IL6R in gastric adenocarcinoma, to promote the proliferative potential of tumor cells [25]. The inhibition of miR-23a by antisense oligonucleotide, inhibited proliferation and promoted apoptosis of the gastric adenocarcinoma cells [75]. The up-regulation of the miR-23a, inhibited the gastric cancer cell apoptosis by down-regulating the Programmed Cell Death 4 (PDCD4). Inhibition of the miR-23a expression in gastric cancer cells, promoted cellular apoptosis [27]. Liu et al. identified Metallothionein 2A (MT2A) as the target of the miR-23a in gastric cancer and MT2A suppression may mediate the promoting effect of miR-23a on the gastric cancer progression [76]. This was similarly observed by An et al. [30]. 

Yong et al. reported that APAF-1 is the direct target of miR-23a in colon cancer cells. miR-23a-suppressed APAF-1 led to the inhibition of apoptosis in colorectal cancer, whereas, the inhibition of miR-23a promoted cell death [23]. A similar observation was reported in pancreatic ductal adenocarcinoma [77], glioma [34], and laryngeal cancer [78].

The suppression of miR-23a in the HCC cells reduced their proliferation but failed to affect cell apoptosis [37]. A further study by Yan et al. showed that miR-23a might mediate the ability of the HCC cells to evade programmed cell death. miR-23a may directly target the 3′UTR of Interferon Regulatory Factor 1 (IRF-1) mRNA to suppress protein expression [79]. However, another study suggested that, in the HCC cells, the miR-23a expression is inversely correlated with the X-linked inhibitor of the apoptosis protein (XIAP). Decreased XIAP, in response to the miR-23a up-regulation, contributed to the caspase-3 activation and cell apoptosis. The up-regulation of the miR-23a was dependent on p53, which was activated in response to estrogen, suggesting a role for the p53-miR-23a/XIAP axis in mediating estrogen-inhibited HCC development [80].

miR-23 suppresses the expression of ST7L tumor suppressor in ovarian cancer cells by directly binding to the 3′UTR of its mRNA, thereby, promoting cell cycle progression and repressing apoptosis [45].

Other reported targets of the miR-23a to promote cancer cell proliferation, while suppressing cellular apoptosis, include phosphatase and tensin homolog (PTEN) [81], special AT-Rich Sequence Binding Protein 1 (SATB1) [17], Fas [39], protein Phosphatase 2 Regulatory Subunit B’Epsilon (PPP2R5E), and Raf kinase inhibitor protein (RKIP) [8].

### 3.3. Cancer Cell Migration and Invasion

**Figure 3:**
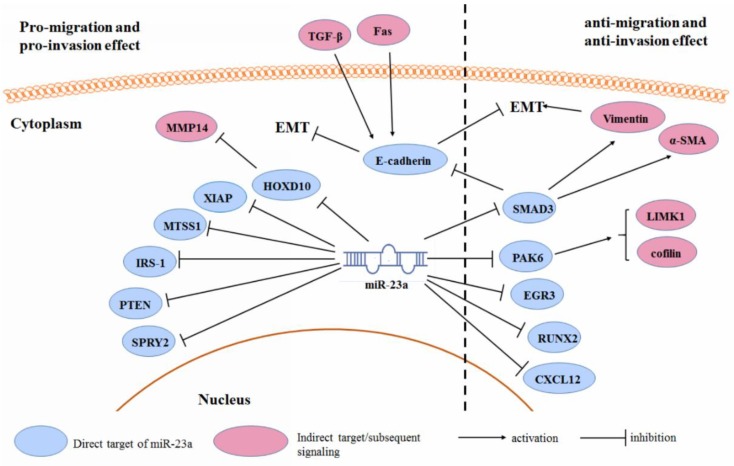
Regulation of the miR-23a on cancer cell migration and invasion.

During cancer progression, tumor cells may increase their ability to detach from cell–cell contact and migrate through blood vessels, which is regarded as cancer metastasis in most cases. The process of metastasis requires activation of genes that may be involved in the epithelial-to-mesenchymal transition (EMT), as well as in the degradation of cell junction and extracellular matrix. It has been extensively reported that the miR-23a is one of the critical mediators in regulating gene expression during the migration and invasion of various human cancer cells (Figure 3).

Over-expression of the miR-23a, significantly promotes cell migration and invasion in various cancers, including breast cancer [82], lung cancer [82], colon cancer [49], gastric cancer [83], pancreatic cancer [47], glioma [35], neuroblastoma [84], osteosarcoma [85], laryngeal cancer [40], and renal cell carcinoma [48]. This probably results from the regulation of the EMT-associated gene expression by miR-23a. Ma et al. showed that miR-23a inhibition suppressed the TGF-β1-induced EMT, and therefore, suppressed the migration, invasion, and metastasis of breast cancer cells. A mechanistic study showed that miR-23a could directly target epithelial marker gene *CDH1*, and reduce the expression of the E-cadherin. Inhibition of the E-cadherin by the miR-23a, activated the Wnt/β-catenin signaling, which is responsible for the TGF-β1-associated tumor invasion [52]. This was similarly observed in lung cancer cells [82] and metastatic neuroblastoma [84]. The expression of the miR-23a was up-regulated upon Fas ligand treatment. Fas ligand induces the ERK1/2 pathway, which in turn activates the AP-1 complex and suppresses the GSK-3β pathway. This leads to the nuclear translocation of the AP-1 and NFAT4, to bind with the promoter of the miR-23a, resulting in a gene transcription. Up-regulation of the miR-23a-targeted E-cadherin, mediates the Fas-induced EMT in gastric cancer [83]. Another study suggested that the role of the miR-23a in the TGF-β1-induced EMT might also be associated with Epithelial Splicing Regulatory Protein 1 (ESRP1) expression in pancreatic cancer cells [47]. Expression of the miR-23a may also indirectly target the repressor of the matrix metallopeptidase 14 (MMP14), homeobox D10 (HOXD10), and result in glioma cell migration [35]. Other reported targets of the miR-23a, whose suppression facilitated the cancer cell migration and invasion, include XIAP [86], metastasis suppressor protein 1 (MTSS1) [22,49], insulin receptor substrate 1 (IRS-1) [41], Sprouty homolog 2 (SPRY2) [31,87], and PTEN [85].

Controversially, several studies observed that miR-23a might be the repressor of tumor cell migration and invasion. In ESCC, miR-23a may increase E-cadherin and reduce vimentin and α-SMA, by down-regulating the SMAD3, which results in a reversal of the EMT [14]. miR-23a expression suppressed the migration and invasion of prostate cancer, through a PAK6 inhibition, by binding to its 3′UTR. This led to the inhibition of the phosphorylation of LIM kinase-1 (LIMK1) and cofilin, which in turn suppressed the formation of stress fibers and actin filaments [13]. miR-23a was found to counteract the migration and invasion of lung cancer cells. This is related to the regulation of the miR-23a on the EGR3 mRNA expression. The 3′UTR of the EGR3 mRNA was found to be the direct target of the miR-23a [88]. In the osteosarcoma cells, an ectopic expression of the miR-23a, led to the retarded migration and invasion of tumor cells. This is associated with the reduced expression of Runt-related transcription factor 2 (RUNX2) and C-X-C motif chemokine 12 (CXCL12) in tumor cells [16].

Furthermore, it is noteworthy that the role of the miR-23a as a non-invasive marker of human cancer diagnosis, as aforementioned, cannot explain its function as a mediator of cancer migration and invasion. The controversial results between the two aspects of the miR-23a suggest that the functional roles of the miR-23a in different types of cancer, may be context-dependent.

### 3.4. Cancer Metabolism

**Figure 4:**
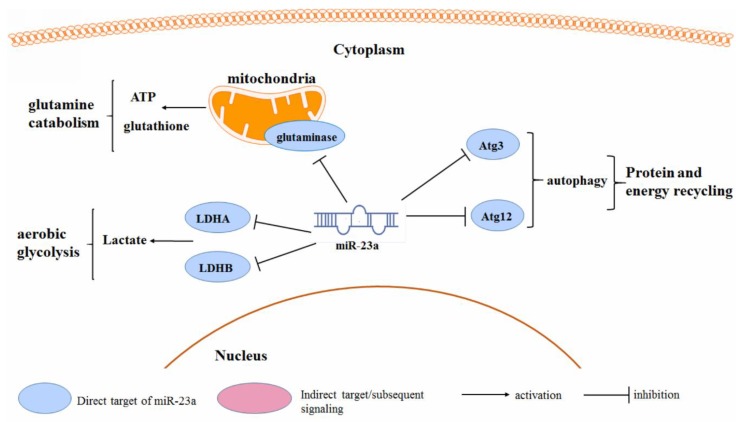
Regulation of miR-23a on the cancer cell metabolism.

In order to maintain the energy requirement of rapid growth, cancer cells have unique features in glucose, amino acid, and fatty acid metabolism. Several studies have suggested that the miR-23a may play a role in the metabolic switch of tumor cells (Figure 4). Saumet et al. identified that miR-23a may be involved in the aerobic glycolysis in breast cancer cells. miR-23a can directly target the 3′UTR of Lactate dehydrogenase (LDH) A and B, the essential enzymes mediating lactate production, during the aerobic glycolysis of breast cancer cells [89]. miR-23a was found to mediate the endoplasmic reticulum (ER) stress-induced metabolic switch, in ovarian cancer cells. miR-23a expression is repressed during ER stress, which led to the induced expression and activity of aerobic glycolytic enzymes LDHA and LDHB. LDHs were identified as the direct target of the miR-23a. While it was found that oncogenic c-Myc can repress the miR-23a transcription, this finding suggests an important role of the miR-23a in the c-Myc-regulated reprograming of the glucose metabolism in cancer [90]. Mitochondrial glutaminase was found to be the direct target of the miR-23a in human P-493 B lymphoma cells and PC3 prostate cancer cells. The inhibition of mitochondrial glutaminase by miR-23a, impaired the glutamine catabolism, which is important for the glutamine-mediated ATP production and glutathione synthesis. The c-Myc-induced miR-23a inhibition enhanced the glutaminase isoform GLS expression, and initiated the metabolic reprograming of tumor cells towards a glutamate-addictive phenotype [91]. A similar observation was reported in the leukemic Jurat cells, in which the over-expression of miR-23a, impaired the glutamine use and induced mitochondrial dysfunction-associated cell death. Glutamine can activate the p65 NF-κB subunit, which binds to the miR-23a promoter as a transcription repressor, to suppress the miR-23a expression. This supports the claim that the decreased expression of the miR-23a facilitates the leukemic cells in glutamine consumption as an alternative source of carbon, and favors their adaptation to the metabolic environment [92]. Gu et al. reported that the miR-23a targets autophagy-associated protein Atg3 and suppresses autophagy in breast cancer cells [93]. Another study suggested that the miR-23a blocked the Atg12 expression in melanoma cells, which in turn reduced the cellular ATP levels. The lack of cellular ATP, activated the AMPK/ACC pathway to block the migration and invasion of tumor cells [18].

### 3.5. Radio- and Chemo-Resistance

**Figure 5:**
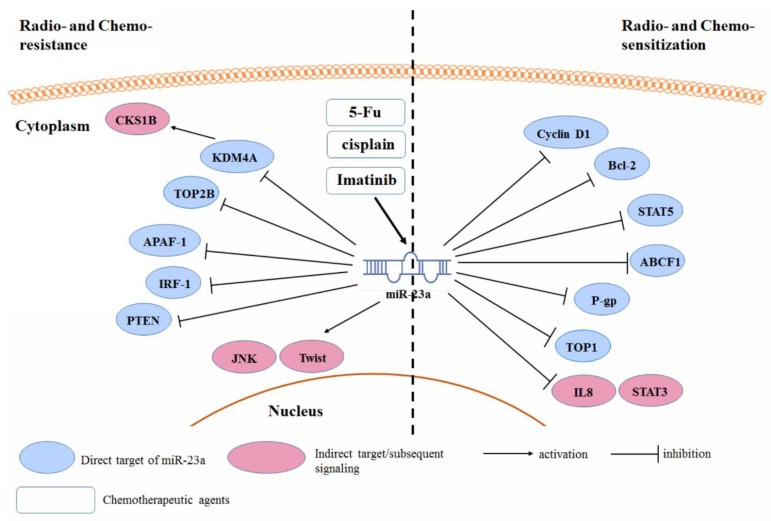
Regulation of the miR-23a on the sensitivity of cancer cells to the anti-cancer treatment.

In addition to the regulation of the oncogenic property of cancer cells, miR-23a was frequently observed to be responsible for the sensitivity of cancer cells to anti-cancer treatment (Figure 5). In some cases, miR-23a may be induced or suppressed upon provision of anti-cancer treatment, such as doxorubicin [94], which in turn regulates the relevant cellular signaling responsible for the sensitivity of cancer cells. For instance, imatinib induced a miR-23a expression, which is involved in the regulation of the sensitivity of chronic myeloid leukemia cells to imatinib. The induced expression of the miR-23a binds to the 3’UTRs of *STAT5*, *CCND1*, and *Bcl-2* genes in the K562 cells, and therefore, increases the cell apoptosis [95]. In contrast, the acquired expression of the miR-23a by anti-cancer treatment was found to reduce the sensitivity of cancer cells. In colon cancer cells, 5-Fu treatment induces miR-23a expression. This contributed to the chemoresistance of the colon cancer cells to the 5-Fu, as the increased expression of the miR-23a can target the APAF-1 to reduce the cell apoptosis. miR-23a antisense sensitized the colon cancer cells to the 5-Fu [96]. Li et al. also showed that the down-regulation of the miR-23a reduced the cell viability and provoked apoptosis in the 5-Fu-treated colorectal cancer cells. ABCF1 was found to be the direct target of the miR-23a and was associated with the chemosensitivity of the colon cancer cells to the 5-Fu [97]. miR-23a expression increased during the acquisition of chemoresistance of tongue squamous carcinoma cells against cisplatin, which may be associated with the miR-23a-mediated TOP2B suppression [98]. A similar expression of the miR-23a-induced chemoresistance, to both 5-Fu and cisplatin, was also observed in the ESCC cells [57].

Some other studies suggested that miR-23a may regulate the sensitivity of cancer cells, although the anti-cancer treatment might not change its endogenous expression. In this case, the regulation of the miR-23a expression in cancer cells is achieved by the ectopic activation/suppression. Over-expression of the miR-23a in tongue squamous cell carcinoma induces Twist expression, JNK activity, and was found to reduce the chemosensitivity of tumor cells toward the cisplatin treatment [99]. Another study suggested that the drug-resistant oncogene *CKS1B* is responsible for the resistance of breast cancer cells to the cisplatin treatment. Breast cancer cells over-expressed CKS1B by regulating the miR-23a-induced suppression of the histone demethylase KDM4A [100]. In gastric adenocarcinoma, the over-expression of the miR-23a reduced the apoptosis and proliferation inhibition induced by paclitaxel. This could be related to the reduction of the IRF1, which is a direct target of the miR-23a. Restoration of the IRF1 counteracts the chemoresistance of tumor cells induced by the miR-23a [101]. Lung cancer cells that over-expressed miR-23a was found to be more resistant to the gefitinib treatment [82]. Lung cancer stem cells (CSCs) exhibited significant resistance to the erlotinib treatment. This could be due to the aberrant expression of the miR-23a and a down-regulation of the PTEN in the CSCs, compared to non-CSCs. As the direct target of the miR-23a, PTEN inhibition led to the reduced response of the PI3K/Akt pathway to the erlotinib treatment in lung CSCs [102]. miR-23a was found to regulate the radiosensitivity of the NPC cells. In radioresistant NPC cells, the expression of the miR-23a is often down-regulated, and the over-expression of the miR-23a potentiates the NPC cells toward radiotherapy. This may be related to the activity of the IL8/Stat3 signaling in the NPC cells. IL-8 and phosphorylated-Stat3 levels are inversely associated with the miR-23a, in the radioresistant NPC tissue. However, no direct target of the miR-23a, in the NPC cells has been identified as a radioresistance-associated gene [69]. In contrast, in ovarian cancer cells, the inhibition of miR-23a, improved the chemosensitivity of tumor cells toward the cisplatin treatment. Reduced miR-23a promotes the cisplatin-induced G0/G1 cell cycle arrest and apoptosis. This may be related to the reduced expression of the multidrug-resistant protein P-gp [103,104]. Wang et al. observed that the over-expression of the miR-23a notably potentiated the HCC cells toward the TOP2A inhibitor etoposide, whereas, it had a minimal effect on the response to the 5-Fu [7]. TOP1 was identified as the direct target of the miR-23a, and the suppression of the TOP1 by miR-23a, led to a cellular topoisomerase activity that fell below the crucial threshold and triggered cell death. The DNA damage induced the expression of the miR-23a in the HCC cells, which was dependent on the p53 up-regulation and activation, during the DNA double-strand breakdown. Suppression of the p53 inhibited the miR-23a pri-, pre-, and mature expression in the HCC cells [7].

### 3.6. Regulation of the Tumor Microenvironment

**Figure 6:**
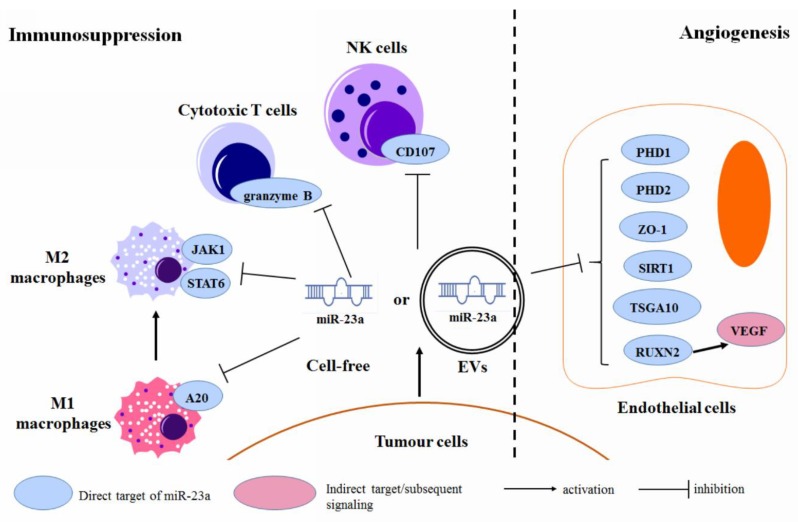
Regulation of the miR-23a on the microenvironment of human cancer.

The research focusing on the microenvironment of human cancer has significantly progressed, suggesting that the tumor microenvironment plays a key role in regulating cancer initiation, progression, metastasis, as well as drug response [105]. Cell and non-cell components within the tumor microenvironment are involved in cancer development via various mechanisms—an idea that has been well-reviewed elsewhere. miR-23a, in the form of cell-free small RNA itself and as a component of the tumor-cell-derived microvesicles (MVs), was found to modulate the properties of the immune and vascular endothelial cells, within the tumor microenvironment (Figure 6). The specific mechanism of how MVs pack and deliver the miR-23a is not clear, yet, but it is generally considered to share a common machinery with the other exosomal miRNAs [106]. It is also not clear if the serum miR-23a is the same as the MVs/exosomes-incorporated miR-23a, as there is no study to distinguish between the two sources of the plasma miR-23a, but in general, plasma miRNAs could be contributed by both MVs/exosomes-incorporated and cell-free miRNAs. A previous study, however, showed that in prostate cancer patients, the expression profiles of miRNAs from these two fractions were totally different [107]. Details about circulating cell-free and MVs/exosome-incorporated miR-23a needs further investigation.

Ma et al. showed that miR-23a might modulate the tumor microenvironment of breast cancer by targeting the Tumor Associated Macrophages (TAMs) [108]. They found that, in M1-like TAMs, miR-23a expression was transcriptionally activated by the p65 nuclear factor-κB (NF-κB), which in turn targets A20 in the NF-κB pathway, to promote the production of pro-inflammatory cytokine; in M2-like macrophages, Stat6 occupies the miR-23a promoter to activate its transcription, and miR-23a in the M2-like TAMs suppress the JAK1/Stat6 pathway, by directly targeting the JAK1 and Stat6. In this case, a reduced expression of the miR-23a in breast cancer, led to an immunosuppressive tumor microenvironment, which facilitated the cancer progression [108]. In patients with advanced lung cancer, miR-23a expression was significantly up-regulated in tumor-infiltrating cytotoxic T lymphocytes (CTLs). This involves the interaction between the tumor-cell-derived TGF-β and CTLs. Elevating the miR-23a blocked the granzyme B expression and, thereby, suppressed the CTL immune function, in advanced lung cancer [109]. Berchem et al. found that the hypoxic-tumor cell-derived MVs were qualitatively different from those in the normoxic tumor cells. Hypoxic MVs enriched the miR-23a, which targeted the CD107a in NK cells, within the tumor microenvironment, and impaired the NK cell function and further led to an immunosuppressive environment in cancer [110].

Lu et al. identified that the miR-23a in MVs secreted by acute lymphocytic leukemia (ALL) cells regulated the expression of zinc finger protein 267 (ZNF267), an oncogenic factor [111]. This further led to the alteration of the cellular signaling process involved in proliferation, differentiation, apoptosis, and cell cycle regulation. miR-23a was found to be significantly enriched in the exosome from the lung cancer cells, with the mesenchymal phenotype [112]. These tumor-cell-derived exosomes may carry the miR-23a to the endothelial cells in lung cancer. Exosomal miR-23a may directly target PHD1 and PHD2 in the endothelial cells, within the tumor microenvironment, which further induces the accumulation of hypoxia-inducible factor (HIF)-1α and promotes tumor angiogenesis. Exosomal miR-23a also suppressed the Zonula occludens-1 (ZO-1) expression in endothelial cells, to increase the vascular permeability and trans-endothelial migration of cancer cells [113]. Another study suggested that the extracellular vesicles (EVs) secreted by lung cancer cells, enriched the miR-23a, whose engulfment led to the down-regulation of the PTEN. Reduced PTEN was then associated with EV-induced angiogenesis in lung cancer [114]. Sruthi et al. further suggested that SIRT1 in endothelial cells is the direct target of the exosomal miR-23a, derived from lung cancer cells, and the down-regulation of the SIRT1 by the exosomal miR-23a, promoted the angiogenesis of lung cancer [115]. Bao et al. suggested that exosomal miR-23a might facilitate an NPC angiogenesis by targeting TSGA10 in the vascular endothelial cells of the NPC microenvironment. Reduced TSGA10 expression led to the growth, migration, and tube formation of the vascular endothelial cells [50]. Wu et al. showed that miR-23a might also suppress the expression of the vascular endothelial growth factor A (VEGF-A), by targeting its translational facilitator RUNX2 in the vascular endothelial cells [116].

## 4. Regulation of the miR-23a Expression in Human Cancer

Although miR-23a plays multiple roles in the initiation, progression, metastasis, as well as treatment response of human cancer, its expression could be primarily dysregulated by various risk factors of cancer, such as viruses, cytokines, and carcinogenic chemicals. For example, cellular miR-23a in lymphoid and epithelial cancer cells could be induced upon type III versus type I Epstein–Barr virus (EBV) latency [117], whereas in the HPV-positive cancer cells, the miR-23a expression is down-regulated by E6/E7 silencing [118]. TGF-β1 can induce the expression of miR-23a in breast cancer cells [52], HCC [36], and lung cancer cells [119], whereas BMP4 can induce the expression of miR-23a in breast cancer cells [120]. Along with this, endogenous chemicals and foreign compounds, such as retinoic acid [89], osthole [121], andrographolide [122], as well as the Chinese medicinal herb Coptis and its active component berberine [123,124], may differentially regulate miR-23a in cancer cells. Mechanistically, the expression of miR-23a in cancer cells can be regulated through both transcriptional factors and epigenetic factor-dependent mechanisms.

### 4.1. Transcriptional Factors

Various transcriptional activators or repressors have been found to be involved in the regulation of miR-23a expression in cancer, which have been reviewed, as aforementioned, in this study. The regulation of the miR-23a expression by several transcriptional factors, such p53 [7,80], AP-1 [83], c-Myc [90,91], and NF-κB [92], have suggested some underlying mechanisms of the oncogenic or tumor suppressive role of these transcriptional factors, in cancer. Regulation of the miR-23a expression by a particular transcriptional factor could be significantly deviated in different types of cancer. For example, p65 NF-κB can strongly bind to the promoter region of the miR-23a-27a-24 cluster and up-regulate the miR-23a expression in the erythroleukaemia cells [125], and an NF-κB inhibitor 1′-Acetoxychavicol acetate could significantly down-regulate the expression of the miR-23a in the head and neck squamous cell carcinoma cells [81]. However, the p65 NF-κB subunit could be a transcriptional repressor of the miR-23a, in leukemic Jurkat cells. This controversy was also found in the c-Myc-regulating miR-23a transcription [87].

Transcriptional factors-regulated miR-23a expression could be induced by the environmental stress on tumor cells. In leukemia cells undergoing oxidative stress, oxidative phosphorylation induced the expression of NQO-1 and HO-1, and activated the MAPK ERK5. The activation of the ERK5, initiated the binding of the transcription factor MEF2 to the promoter region of the miR-23a-27a-24-2 cluster, to generate new miR-23a. miR-23a directly targets the KEAP1 mRNA and suppresses its expression [126].

Other reported transcriptional activators of the miR-23a include the cAMP response element binding (CREB) protein [127], Leucine-rich repeat-containing G-protein coupled receptor 5 (LGR5) [128], and Ephrin type-B receptor 6 (EphB6) [129], whereas, its repressors include the zinc finger protein 1 (ZIC1) [43], and the KH-type splicing regulatory protein (KSRP) [88].

### 4.2. Epigenetic Factors

Epigenetic factors have also been reported to mediate the transcriptional and translational machinery of the miR-23a. Among various epigenetic factors, methylation of DNA and lncRNAs were mostly reported to regulate the miR-23a expression.

In K562 cells, the promoter region of the miR-23a is hypermethylated. Treating the K562 cells with a DNA methylation inhibitor 5-aza-2′deoxycytidine, induced the expression of miR-23a [10]. A similar observation was reported in the HCC [130] and osteosarcoma cells [16]. In contrast, Wang et al. identified that the SP1 sites in the promoter region of the miR-23a-27a-24-2 cluster were demethylated, which led to the up-regulation of the miRNAs. A methyl donor, S-adenosyl-L-methionine, could significantly block the binding of SP1 to the gene cluster promoter and suppress the miR-23a expression [131].

An lncRNA GAS5 was found to be suppressed in breast cancer tissue and cell lines, which was associated with larger tumor size, advanced metastasis, and poorer clinical phenotype of breast cancer. GAS5 suppresses the expression of the miR-23a with a sponge mechanism [93]. The regulation of the GAS5 on miR-23a, was also observed in gastric cancer [76] and lung cancer [132]. Another lncRNA X-inactive specific transcript (XIST), negatively regulated the expression of miR-23a, in prostate cancer. This was correlated with poor prognosis, cellular proliferation, and metastasis, in prostate cancer, resulting from a competition between the RKIP mRNA and the miR-23a, for the 3’UTR binding site [133].

## 5. Discussion

Several studies have proposed opposite functions of the miR-23a, even when they studied the same type of cancer. This could be probably due to the inconsistent quality of the studies. For example, in the gain- and loss-of-function design, the use of miR-23a inhibitor and mimics should be further justified. The reference sequence of the products, the efficiency of transfection, and the validation of the miRNAs function were often missed in the reviewed studies. Appropriate positive and negative controls were not available, which would largely affect the consistency of the data. Some guidelines have been recommended for quality control, in miRNA studies [134,135,136], which should be applied in future investigations of miR-23a, in order to produce consistent and reliable conclusions.

While the biological function of miR-23a can be further investigated by experimental models, its perspective in clinical application remains unclear. This is not only due to the foreseeable general obstacles met in the application of gene therapy, but also the uncertain adverse effect of the miR-23a, as a system. Attempts to use miRNAs, as gene therapy in cancer treatment, remain rare, and unfortunately, the only two registered clinical trials using the miR-Rx34 (NCT02862145 and NCT01829971) have been terminated or withdrawn, due to serious immune-related adverse events. The dim prospect of using miRNAs in the clinical treatment of cancer, at this moment, suggest that more pre-clinical studies, particularly, regarding toxicity and safety, should be performed.

## 6. Conclusions

This review highlighted the scientific achievements in the study of miR-23a in human cancer and outlined the biological and clinical insights on the advancements and challenges of miR-23a as a diagnostic and therapeutic tool in cancer management. miR-23a has been detected as being differentially expressed across different types of cancer, and might predict the survival and treatment response of cancer patients. Its biological functions covered the scenarios of carcinogenesis, cancer progression, metastasis, and drug resistance, suggesting that it might have potential as an emerging targetable entity in cancer treatment.

## Figures and Tables

**Table 1:** Expression pattern of microRNA-23a (miR-23a) in different types of human cancer.

Type of Cancer	References
**Down-Regulated**	
Acute myelogenous leukemia	[8]
Chronic myelogenous leukemia	[9,10]
Endometrial endometrioid adenocarcinoma	[14]
Melanoma	[18]
Nephroblastoma	[15]
Osteosarcoma	[16,17]
Prostate carcinoma	[12,13]
**Up-Regulated**	
Bladder cancer	[19]
Breast cancer	[20]
Cholangiocarcinoma	[21]
Colorectal cancer	[22,23]
Esophageal squamous cell cancer (ESCC)	[24]
Gastric carcinoma	[25,26,27,28,29,30,31]
Glioblastoma & glioma	[32,33,34,35]
Hepatocellular carcinoma	[36,37]
Large B-cell lymphoma	[38,39]
Laryngeal cancer	[40]
Lung carcinoma	[41,42]
Malignant pleural mesothelioma (MPM)	[43]
Ovarian carcinoma	[44,45]
Pancreatic cancer	[46,47]
Renal cell carcinoma (RCC)	[48]

## References

[1] Pekarsky Y., Balatti V., Croce C.M. (2018). BCL2 and miR-15/16: From gene discovery to treatment. Cell Death Differ..

[2] Vidigal J.A., Ventura A. (2015). The biological functions of miRNAs: Lessons from in vivo studies. Trends Cell Biol..

[3] Croce C.M. (2009). Causes and consequences of microRNA dysregulation in cancer. Nat. Rev. Genet..

[4] Acunzo M., Romano G., Wernicke D., Croce C.M. (2015). MicroRNA and cancer—A brief overview. Adv. Biol. Regul..

[5] Kurkewich J.L., Hansen J., Klopfenstein N., Zhang H., Wood C., Boucher A., Hickman J., Muench D.E., Grimes H.L., Dahl R. (2017). The miR-23a~27a~24-2 microRNA cluster buffers transcription and signaling pathways during hematopoiesis. PLoS Genet..

[6] Zhang Y., Peng B., Han Y. (2018). MiR-23a regulates the proliferation and migration of human pulmonary artery smooth muscle cells (HPASMCs) through targeting BMPR2/Smad1 signaling. Biomed. Pharmacother..

[7] Wang N., Zhu M., Tsao S.W., Man K., Zhang Z., Feng Y. (2013). MiR-23a-mediated inhibition of topoisomerase 1 expression potentiates cell response to etoposide in human hepatocellular carcinoma. Mol. Cancer.

[8] Hatzl S., Geiger O., Kuepper M.K., Caraffini V., Seime T., Furlan T., Nussbaumer E., Wieser R., Pichler M., Scheideler M. (2016). Increased Expression of miR-23a Mediates a Loss of Expression in the RAF Kinase Inhibitor Protein RKIP. Cancer Res..

[9] Zhu X., Lin Z., Du J., Zhou X., Yang L., Liu G. (2014). Studies on microRNAs that are correlated with the cancer stem cells in chronic myeloid leukemia. Mol. Cell. Biochem..

[10] Xishan Z., Xianjun L., Ziying L., Guangxin C., Gang L. (2014). The malignancy suppression role of miR-23a by targeting the BCR/ABL oncogene in chromic myeloid leukemia. Cancer Gene Ther..

[11] Su R., Dong L., Zou D., Zhao H., Ren Y., Li F., Yi P., Li L., Zhu Y., Ma Y. (2016). microRNA-23a, -27a and -24 synergistically regulate JAK1/Stat3 cascade and serve as novel therapeutic targets in human acute erythroid leukemia. Oncogene.

[12] Aghaee-Bakhtiari S.H., Arefian E., Naderi M., Noorbakhsh F., Nodouzi V., Asgari M., Fard-Esfahani P., Mahdian R., Soleimani M. (2015). MAPK and JAK/STAT pathways targeted by miR-23a and miR-23b in prostate cancer: Computational and in vitro approaches. Tumour Biol..

[13] Cai S., Chen R., Li X., Cai Y., Ye Z., Li S., Li J., Huang H., Peng S., Wang J. (2015). Downregulation of microRNA-23a suppresses prostate cancer metastasis by targeting the PAK6-LIMK1 signaling pathway. Oncotarget.

[14] Liu P., Wang C., Ma C., Wu Q., Zhang W., Lao G. (2016). MicroRNA-23a regulates epithelial-to-mesenchymal transition in endometrial endometrioid adenocarcinoma by targeting SMAD3. Cancer Cell Int..

[15] Koller K., Das S., Leuschner I., Korbelius M., Hoefler G., Guertl B. (2013). Identification of the transcription factor HOXB4 as a novel target of miR-23a. Genes Chromosom. Cancer.

[16] He Y., Meng C., Shao Z., Wang H., Yang S. (2014). MiR-23a functions as a tumor suppressor in osteosarcoma. Cell. Physiol. Biochem..

[17] Wang G., Li B., Fu Y., He M., Wang J., Shen P., Bai L. (2015). miR-23a suppresses proliferation of osteosarcoma cells by targeting SATB1. Tumour Biol..

[18] Guo W., Wang H., Yang Y., Guo S., Zhang W., Liu Y., Yi X., Ma J., Zhao T., Liu L. (2017). Down-regulated miR-23a Contributes to the Metastasis of Cutaneous Melanoma by Promoting Autophagy. Theranostics.

[19] Gottardo F., Liu C.G., Ferracin M., Calin G.A., Fassan M., Bassi P., Sevignani C., Byrne D., Negrini M., Pagano F. (2007). Micro-RNA profiling in kidney and bladder cancers. Urol. Oncol..

[20] Eissa S., Matboli M., Shehata H.H. (2015). Breast tissue-based microRNA panel highlights microRNA-23a and selected target genes as putative biomarkers for breast cancer. Trans. Res..

[21] Meng F., Henson R., Lang M., Wehbe H., Maheshwari S., Mendell J.T., Jiang J., Schmittgen T.D., Patel T. (2006). Involvement of human micro-RNA in growth and response to chemotherapy in human cholangiocarcinoma cell lines. Gastroenterology.

[22] Jahid S., Sun J., Edwards R.A., Dizon D., Panarelli N.C., Milsom J.W., Sikandar S.S., Gumus Z.H., Lipkin S.M. (2012). miR-23a promotes the transition from indolent to invasive colorectal cancer. Cancer Discov..

[23] Yong F.L., Wang C.W., Roslani A.C., Law C.W. (2014). The involvement of miR-23a/APAF1 regulation axis in colorectal cancer. Int. J. Mol. Sci..

[24] Zhu L., Jin L., Jiang R., Wang Q., Jiang J., Mao C., Chen D. (2013). Correlations between miRNAs and TGF-β1 in tumor microenvironment of esophageal squamous cell cancer. Chin J. Cell. Mol. Immunol..

[25] Zhu L.H., Liu T., Tang H., Tian R.Q., Su C., Liu M., Li X. (2010). MicroRNA-23a promotes the growth of gastric adenocarcinoma cell line MGC803 and downregulates interleukin-6 receptor. FEBS J..

[26] Ma G., Dai W., Sang A., Yang X., Gao C. (2014). Upregulation of microRNA-23a/b promotes tumor progression and confers poor prognosis in patients with gastric cancer. Int. J. Clin. Exp. Pathol..

[27] Hu X., Wang Y., Liang H., Fan Q., Zhu R., Cui J., Zhang W., Zen K., Zhang C.Y., Hou D. (2017). miR-23a/b promote tumor growth and suppress apoptosis by targeting PDCD4 in gastric cancer. Cell Death Dis..

[28] Hua K., Chen Y.T., Chen C.F., Tang Y.S., Huang T.T., Lin Y.C., Yeh T.S., Huang K.H., Lee H.C., Hsu M.T. (2018). MicroRNA-23a/27a/24-2 cluster promotes gastric cancer cell proliferation synergistically. Oncol. Lett..

[29] Li X., Zhang Y., Zhang H., Liu X., Gong T., Li M., Sun L., Ji G., Shi Y., Han Z. (2011). miRNA-223 promotes gastric cancer invasion and metastasis by targeting tumor suppressor EPB41L3. Mol. Cancer Res..

[30] An J., Pan Y., Yan Z., Li W., Cui J., Yuan J., Tian L., Xing R., Lu Y. (2013). MiR-23a in amplified 19p13.13 loci targets metallothionein 2A and promotes growth in gastric cancer cells. J. Cell. Biochem..

[31] Li Y., Chen H., She P., Chen T., Chen L., Yuan J., Jiang B. (2018). microRNA-23a promotes cell growth and metastasis in gastric cancer via targeting SPRY2-mediated ERK signaling. Oncol. Lett..

[32] Rao S.A., Santosh V., Somasundaram K. (2010). Genome-wide expression profiling identifies deregulated miRNAs in malignant astrocytoma. Mod. Pathol..

[33] Koshkin P.A., Chistiakov D.A., Nikitin A.G., Konovalov A.N., Potapov A.A., Usachev D.Y., Pitskhelauri D.I., Kobyakov G.L., Shishkina L.V., Chekhonin V.P. (2014). Analysis of expression of microRNAs and genes involved in the control of key signaling mechanisms that support or inhibit development of brain tumors of different grades. Clin. Chim. Acta.

[34] Lian S., Shi R., Bai T., Liu Y., Miao W., Wang H., Liu X., Fan Y. (2013). Anti-miRNA-23a oligonucleotide suppresses glioma cells growth by targeting apoptotic protease activating factor-1. Curr. Pharm. Des..

[35] Hu X., Chen D., Cui Y., Li Z., Huang J. (2013). Targeting microRNA-23a to inhibit glioma cell invasion via HOXD10. Sci. Rep..

[36] Huang S., He X., Ding J., Liang L., Zhao Y., Zhang Z., Yao X., Pan Z., Zhang P., Li J. (2008). Upregulation of miR-23a approximately 27a approximately 24 decreases transforming growth factor-beta-induced tumor-suppressive activities in human hepatocellular carcinoma cells. Int. J. Cancer.

[37] Bao L., Zhao J., Dai X., Wang Y., Ma R., Su Y., Cui H., Niu J., Bai S., Xiao Z. (2014). Correlation between miR-23a and onset of hepatocellular carcinoma. Clin. Res. Hepatal. Gastroenterol..

[38] Wang W.L., Yang C., Han X.L., Wang R., Huang Y., Zi Y.M., Li J.D. (2014). MicroRNA-23a expression in paraffin-embedded specimen correlates with overall survival of diffuse large B-cell lymphoma. Med. Oncol..

[39] Li B., Sun M., Gao F., Liu W., Yang Y., Liu H., Cheng Y., Liu C., Cai J. (2013). Up-regulated expression of miR-23a/b targeted the pro-apoptotic Fas in radiation-induced thymic lymphoma. Cell. Physiol. Biochem..

[40] Zhang X.W., Liu N., Chen S., Wang Y., Zhang Z.X., Sun Y.Y., Qiu G.B., Fu W.N. (2015). High microRNA-23a expression in laryngeal squamous cell carcinoma is associated with poor patient prognosis. Diagn. Pathol..

[41] Cao M., Li Y., Lu H., Meng Q., Wang L., Cai L., Dong X. (2014). MiR-23a-mediated migration/invasion is rescued by its target, IRS-1, in non-small cell lung cancer cells. J. Cancer Res. Clin. Oncol..

[42] Qu W.Q., Liu L., Yu Z. (2015). Clinical value of microRNA-23a upregulation in non-small cell lung cancer. Int. J. Clin. Exp. Med..

[43] Cheng Y.Y., Kirschner M.B., Cheng N.C., Gattani S., Klebe S., Edelman J.J., Vallely M.P., McCaughan B.C., Jin H.C., van Zandwijk N. (2013). ZIC1 is silenced and has tumor suppressor function in malignant pleural mesothelioma. J. Thorac. Oncol..

[44] Vaksman O., Stavnes H.T., Kaern J., Trope C.G., Davidson B., Reich R. (2011). miRNA profiling along tumour progression in ovarian carcinoma. J. Cell. Mol. Med..

[45] Yang Z., Wang X.L., Bai R., Liu W.Y., Li X., Liu M., Tang H. (2016). miR-23a promotes IKKalpha expression but suppresses ST7L expression to contribute to the malignancy of epithelial ovarian cancer cells. Br. J. Cancer.

[46] Piepoli A., Tavano F., Copetti M., Mazza T., Palumbo O., Panza A., di Mola F.F., Pazienza V., Mazzoccoli G., Biscaglia G. (2012). Mirna expression profiles identify drivers in colorectal and pancreatic cancers. PLoS ONE.

[47] Wu G., Li Z., Jiang P., Zhang X., Xu Y., Chen K., Li X. (2017). MicroRNA-23a promotes pancreatic cancer metastasis by targeting epithelial splicing regulator protein 1. Oncotarget.

[48] Quan J., Jin L., Pan X., He T., Lai Y., Chen P., Lin C., Yang S., Zeng H., Lai Y. (2017). Oncogenic miR-23a-5p is associated with cellular function in RCC. Mol. Med. Rep..

[49] Tang H.L., Deng M., Liao Q.J., Zeng X., Zhou X.T., Su Q. (2012). Expression and clinical significance of miR-23a and metastasis suppressor 1 in colon carcinoma. Chin. J. Pathol..

[50] Bao L., You B., Shi S., Shan Y., Zhang Q., Yue H., Zhang J., Zhang W., Shi Y., Liu Y. (2018). Metastasis-associated miR-23a from nasopharyngeal carcinoma-derived exosomes mediates angiogenesis by repressing a novel target gene TSGA10. Oncogene.

[51] Su L., Liu M. (2018). Correlation analysis on the expression levels of microRNA-23a and microRNA-23b and the incidence and prognosis of ovarian cancer. Oncol. Lett..

[52] Ma F., Li W., Liu C., Li W., Yu H., Lei B., Ren Y., Li Z., Pang D., Qian C. (2017). MiR-23a promotes TGF-beta1-induced EMT and tumor metastasis in breast cancer cells by directly targeting CDH1 and activating Wnt/beta-catenin signaling. Oncotarget.

[53] Wu Q., Lu Z., Li H., Lu J., Guo L., Ge Q. (2011). Next-generation sequencing of microRNAs for breast cancer detection. J. Biomed. Biotechnol..

[54] Wu Q., Wang C., Lu Z., Guo L., Ge Q. (2012). Analysis of serum genome-wide microRNAs for breast cancer detection. Clin. Chim. Acta.

[55] Nakatani F., Ferracin M., Manara M.C., Ventura S., Del Monaco V., Ferrari S., Alberghini M., Grilli A., Knuutila S., Schaefer K.L. (2012). miR-34a predicts survival of Ewing’s sarcoma patients and directly influences cell chemo-sensitivity and malignancy. J. Pathol..

[56] Frampton A.E., Castellano L., Colombo T., Giovannetti E., Krell J., Jacob J., Pellegrino L., Roca-Alonso L., Funel N., Gall T.M. (2015). Integrated molecular analysis to investigate the role of microRNAs in pancreatic tumour growth and progression. Lancet.

[57] Komatsu S., Ichikawa D., Kawaguchi T., Takeshita H., Miyamae M., Ohashi T., Okajima W., Imamura T., Kiuchi J., Arita T. (2016). Plasma microRNA profiles: Identification of miR-23a as a novel biomarker for chemoresistance in esophageal squamous cell carcinoma. Oncotarget.

[58] Yang C., Wang C., Chen X., Chen S., Zhang Y., Zhi F., Wang J., Li L., Zhou X., Li N. (2013). Identification of seven serum microRNAs from a genome-wide serum microRNA expression profile as potential noninvasive biomarkers for malignant astrocytomas. Int. J. Cancer.

[59] Cinpolat O., Unal Z.N., Ismi O., Gorur A., Unal M. (2017). Comparison of microRNA profiles between benign and malignant salivary gland tumors in tissue, blood and saliva samples: A prospective, case-control study. Braz. J. Otorhinolaryngol..

[60] Humeau M., Vignolle-Vidoni A., Sicard F., Martins F., Bournet B., Buscail L., Torrisani J., Cordelier P. (2015). Salivary MicroRNA in Pancreatic Cancer Patients. PLoS ONE.

[61] Khare D., Goldschmidt N., Bardugo A., Gur-Wahnon D., Ben-Dov I.Z., Avni B. (2017). Plasma microRNA profiling: Exploring better biomarkers for lymphoma surveillance. PLoS ONE.

[62] Yong F.L., Law C.W., Wang C.W. (2013). Potentiality of a triple microRNA classifier: MiR-193a-3p, miR-23a and miR-338-5p for early detection of colorectal cancer. BMC Cancer.

[63] Ogata-Kawata H., Izumiya M., Kurioka D., Honma Y., Yamada Y., Furuta K., Gunji T., Ohta H., Okamoto H., Sonoda H. (2014). Circulating exosomal microRNAs as biomarkers of colon cancer. PLoS ONE.

[64] Ostenfeld M.S., Jensen S.G., Jeppesen D.K., Christensen L.L., Thorsen S.B., Stenvang J., Hvam M.L., Thomsen A., Mouritzen P., Rasmussen M.H. (2016). miRNA profiling of circulating EpCAM(+) extracellular vesicles: Promising biomarkers of colorectal cancer. J. Extracell. Vesicles.

[65] Vychytilova-Faltejskova P., Radova L., Sachlova M., Kosarova Z., Slaba K., Fabian P., Grolich T., Prochazka V., Kala Z., Svoboda M. (2016). Serum-based microRNA signatures in early diagnosis and prognosis prediction of colon cancer. Carcinogenesis.

[66] Quan J., Liu S., Dai K., Jin L., He T., Pan X., Lai Y. (2018). MicroRNA-23a/24-2/27a as a potential diagnostic biomarker for cancer: A systematic review and meta-analysis. Mol. Clin. Oncol..

[67] Vasaikar S.V., Straub P., Wang J., Zhang B. (2018). LinkedOmics: Analyzing multi-omics data within and across 32 cancer types. Nucleic Acids Res..

[68] El-Halawany M.S., Ismail H.M., Zeeneldin A.A., Elfiky A., Tantawy M., Kobaisi M.H., Hamed I., Abdel Wahab A.H. (2015). Investigating the pretreatment miRNA expression patterns of advanced hepatocellular carcinoma patients in association with response to TACE treatment. Biomed Res. Int..

[69] Qu J.Q., Yi H.M., Ye X., Li L.N., Zhu J.F., Xiao T., Yuan L., Li J.Y., Wang Y.Y., Feng J. (2015). MiR-23a sensitizes nasopharyngeal carcinoma to irradiation by targeting IL-8/Stat3 pathway. Oncotarget.

[70] Ramassone A., Pagotto S., Veronese A., Visone R. (2018). Epigenetics and MicroRNAs in Cancer. Int. J. Mol. Sci..

[71] Tsai Y.S., Lin C.S., Chiang S.L., Lee C.H., Lee K.W., Ko Y.C. (2011). Areca nut induces miR-23a and inhibits repair of DNA double-strand breaks by targeting FANCG. Toxicol. Sci..

[72] Wang B., Hsu S.H., Frankel W., Ghoshal K., Jacob S.T. (2012). Stat3-mediated activation of microRNA-23a suppresses gluconeogenesis in hepatocellular carcinoma by down-regulating glucose-6-phosphatase and peroxisome proliferator-activated receptor gamma, coactivator 1 α. Hepatology.

[73] Ebert M.S., Sharp P.A. (2012). Roles for microRNAs in conferring robustness to biological processes. Cell.

[74] Chen B., Zhu A., Tian L., Xin Y., Liu X., Peng Y., Zhang J., Miao Y., Wei J. (2018). miR23a suppresses pancreatic cancer cell progression by inhibiting PLK1 expression. Mol. Med. Rep..

[75] Liu X., Liu Q., Fan Y., Wang S., Liu X., Zhu L., Liu M., Tang H. (2014). Downregulation of PPP2R5E expression by miR-23a suppresses apoptosis to facilitate the growth of gastric cancer cells. FEBS Lett..

[76] Liu X., Jiao T., Wang Y., Su W., Tang Z., Han C. (2016). Long non-coding RNA GAS5 acts as a molecular sponge to regulate miR-23a in gastric cancer. Int. J. Clin. Exp. Pathol..

[77] Liu N., Sun Y.Y., Zhang X.W., Chen S., Wang Y., Zhang Z.X., Song S.W., Qiu G.B., Fu W.N. (2015). Oncogenic miR-23a in Pancreatic Ductal Adenocarcinogenesis Via Inhibiting APAF1. Dig. Dis. Sci..

[78] Zhang X.W., Liu N., Chen S., Wang Y.E., Sun K.L., Xu Z.M., Fu W.N. (2015). Upregulation of microRNA-23a regulates proliferation and apoptosis by targeting APAF-1 in laryngeal carcinoma. Oncology Lett..

[79] Yan Y., Liang Z., Du Q., Yang M., Geller D.A. (2016). MicroRNA-23a downregulates the expression of interferon regulatory factor-1 in hepatocellular carcinoma cells. Oncol. Rep..

[80] Huang F.Y., Wong D.K., Seto W.K., Lai C.L., Yuen M.F. (2015). Estradiol induces apoptosis via activation of miRNA-23a and p53: Implication for gender difference in liver cancer development. Oncotarget.

[81] Wang H., Shen L., Li X., Sun M. (2013). MicroRNAs contribute to the anticancer effect of 1′-acetoxychavicol acetate in human head and neck squamous cell carcinoma cell line HN4. Biosci. Biotechnol. Biochem..

[82] Cao M., Seike M., Soeno C., Mizutani H., Kitamura K., Minegishi Y., Noro R., Yoshimura A., Cai L., Gemma A. (2012). MiR-23a regulates TGF-β-induced epithelial-mesenchymal transition by targeting E-cadherin in lung cancer cells. Int. J. Oncol..

[83] Zheng H., Li W., Wang Y., Xie T., Cai Y., Wang Z., Jiang B. (2014). miR-23a inhibits E-cadherin expression and is regulated by AP-1 and NFAT4 complex during Fas-induced EMT in gastrointestinal cancer. Carcinogenesis.

[84] Cheng L., Yang T., Kuang Y., Kong B., Yu S., Shu H., Zhou H., Gu J. (2014). MicroRNA-23a promotes neuroblastoma cell metastasis by targeting CDH1. Oncology Lett..

[85] Tian K., Di R., Wang L. (2015). MicroRNA-23a enhances migration and invasion through PTEN in osteosarcoma. Cancer Gene Ther..

[86] Chen P., He Y.H., Huang X., Tao S.Q., Wang X.N., Yan H., Ding K.S., Lobie P.E., Wu W.Y., Wu Z.S. (2017). MiR-23a modulates X-linked inhibitor of apoptosis-mediated autophagy in human luminal breast cancer cell lines. Oncotarget.

[87] Li X., Liu X., Xu W., Zhou P., Gao P., Jiang S., Lobie P.E., Zhu T. (2013). c-MYC-regulated miR-23a/24-2/27a cluster promotes mammary carcinoma cell invasion and hepatic metastasis by targeting Sprouty2. J. Biol. Chem..

[88] Chien M.H., Lee W.J., Yang Y.C., Li Y.L., Chen B.R., Cheng T.Y., Yang P.W., Wang M.Y., Jan Y.H., Lin Y.K. (2017). KSRP suppresses cell invasion and metastasis through miR-23a-mediated EGR3 mRNA degradation in non-small cell lung cancer. Biochim. Biophys. Acta Gene Requl. Mech..

[89] Saumet A., Vetter G., Bouttier M., Antoine E., Roubert C., Orsetti B., Theillet C., Lecellier C.H. (2012). Estrogen and retinoic acid antagonistically regulate several microRNA genes to control aerobic glycolysis in breast cancer cells. Mol. Biosyst..

[90] Poyyakkara A., Raji G.R., Kunhiraman H., Edatt L., Kumar S.V.B. (2018). ER stress mediated regulation of miR23a confer Hela cells better adaptability to utilize glycolytic pathway. J. Cell. Biochem..

[91] Gao P., Tchernyshyov I., Chang T.C., Lee Y.S., Kita K., Ochi T., Zeller K.I., De Marzo A.M., Van Eyk J.E., Mendell J.T. (2009). c-Myc suppression of miR-23a/b enhances mitochondrial glutaminase expression and glutamine metabolism. Nature.

[92] Rathore M.G., Saumet A., Rossi J.F., de Bettignies C., Tempe D., Lecellier C.H., Villalba M. (2012). The NF-kappaB member p65 controls glutamine metabolism through miR-23a. Int. J. Biochem. Cell Biol..

[93] Gu J., Wang Y., Wang X., Zhou D., Wang X., Zhou M., He Z. (2018). Effect of the LncRNA GAS5-MiR-23a-ATG3 Axis in Regulating Autophagy in Patients with Breast Cancer. Cell. Physiol. Biochem..

[94] Rizzo S., Cangemi A., Galvano A., Fanale D., Buscemi S., Ciaccio M., Russo A., Castorina S., Bazan V. (2017). Analysis of miRNA expression profile induced by short term starvation in breast cancer cells treated with doxorubicin. Oncotarget.

[95] Farhadi E., Zaker F., Safa M., Rezvani M.R. (2016). miR-101 sensitizes K562 cell line to imatinib through Jak2 downregulation and inhibition of NF-kappaB target genes. Tumour Biol..

[96] Shang J., Yang F., Wang Y., Wang Y., Xue G., Mei Q., Wang F., Sun S. (2014). MicroRNA-23a antisense enhances 5-fluorouracil chemosensitivity through APAF-1/caspase-9 apoptotic pathway in colorectal cancer cells. J. Cell. Biochem..

[97] Li X., Li X., Liao D., Wang X., Wu Z., Nie J., Bai M., Fu X., Mei Q., Han W. (2015). Elevated microRNA-23a Expression Enhances the Chemoresistance of Colorectal Cancer Cells with Microsatellite Instability to 5-Fluorouracil by Directly Targeting ABCF1. Curr. Protein Pept. Sci..

[98] Yu Z.W., Zhong L.P., Ji T., Zhang P., Chen W.T., Zhang C.P. (2010). MicroRNAs contribute to the chemoresistance of cisplatin in tongue squamous cell carcinoma lines. Oral Oncol..

[99] Peng F., Zhang H., Du Y., Tan P. (2015). miR-23a promotes cisplatin chemoresistance and protects against cisplatin-induced apoptosis in tongue squamous cell carcinoma cells through Twist. Oncol. Rep..

[100] Black J.C., Zhang H., Kim J., Getz G., Whetstine J.R. (2016). Regulation of Transient Site-specific Copy Gain by MicroRNA. J. Biol. Chem..

[101] Liu X., Ru J., Zhang J., Zhu L.H., Liu M., Li X., Tang H. (2013). miR-23a targets interferon regulatory factor 1 and modulates cellular proliferation and paclitaxel-induced apoptosis in gastric adenocarcinoma cells. PLoS ONE.

[102] Han Z., Zhou X., Li S., Qin Y., Chen Y., Liu H. (2017). Inhibition of miR-23a increases the sensitivity of lung cancer stem cells to erlotinib through PTEN/PI3K/Akt pathway. Oncol. Rep..

[103] Jin A.H., Zhou X.P., Zhou F.Z. (2015). Inhibition of microRNA-23a increases cisplatin sensitivity of ovarian cancer cells: The possible molecular mechanisms. J. South. Med. Univ..

[104] Jin A.H., Wei Z.L. (2015). Molecular mechanism of increased sensitivity of cisplatin to ovarian cancer by inhibition of microRNA-23a expression. Int. J. Clin. Exp. Med..

[105] Tan H.Y., Wang N., Lam W., Guo W., Feng Y., Cheng Y.C. (2018). Targeting tumour microenvironment by tyrosine kinase inhibitor. Mol. Cancer.

[106] Zhang J., Li S., Li L., Li M., Guo C., Yao J., Mi S. (2015). Exosome and exosomal microRNA: Trafficking, sorting, and function. Genom. Proteom. Bioinform..

[107] Endzelins E., Berger A., Melne V., Bajo-Santos C., Sobolevska K., Abols A., Rodriguez M., Santare D., Rudnickiha A., Lietuvietis V. (2017). Detection of circulating miRNAs: Comparative analysis of extracellular vesicle-incorporated miRNAs and cell-free miRNAs in whole plasma of prostate cancer patients. BMC Cancer.

[108] Ma S., Liu M., Xu Z., Li Y., Guo H., Ge Y., Liu Y., Zheng D., Shi J. (2016). A double feedback loop mediated by microRNA-23a/27a/24-2 regulates M1 versus M2 macrophage polarization and thus regulates cancer progression. Oncotarget.

[109] Lin R., Chen L., Chen G., Hu C., Jiang S., Sevilla J., Wan Y., Sampson J.H., Zhu B., Li Q.J. (2014). Targeting miR-23a in CD8+ cytotoxic T lymphocytes prevents tumor-dependent immunosuppression. J. Clin. Investig..

[110] Berchem G., Noman M.Z., Bosseler M., Paggetti J., Baconnais S., Le Cam E., Nanbakhsh A., Moussay E., Mami-Chouaib F., Janji B. (2016). Hypoxic tumor-derived microvesicles negatively regulate NK cell function by a mechanism involving TGF-β and miR23a transfer. Oncoimmunology.

[111] Lu L., Chen X.M., Tao H.M., Xiong W., Jie S.H., Li H.Y. (2015). Regulation of the expression of zinc finger protein genes by microRNAs enriched within acute lymphoblastic leukemia-derived microvesicles. Genet. Mol. Res..

[112] Kim J., Kim T.Y., Lee M.S., Mun J.Y., Ihm C., Kim S.A. (2016). Exosome cargo reflects TGF-β1-mediated epithelial-to-mesenchymal transition (EMT) status in A549 human lung adenocarcinoma cells. Biochem. Biophys. Res. Commun..

[113] Hsu Y.L., Hung J.Y., Chang W.A., Lin Y.S., Pan Y.C., Tsai P.H., Wu C.Y., Kuo P.L. (2017). Hypoxic lung cancer-secreted exosomal miR-23a increased angiogenesis and vascular permeability by targeting prolyl hydroxylase and tight junction protein ZO-1. Oncogene.

[114] Zheng Y., Liu L., Chen C., Ming P., Huang Q., Li C., Cao D., Xu X., Ge W. (2017). The extracellular vesicles secreted by lung cancer cells in radiation therapy promote endothelial cell angiogenesis by transferring miR-23a. PeerJ.

[115] Sruthi T.V., Edatt L., Raji G.R., Kunhiraman H., Shankar S.S., Shankar V., Ramachandran V., Poyyakkara A., Kumar S.V.B. (2018). Horizontal transfer of miR-23a from hypoxic tumor cell colonies can induce angiogenesis. J. Cell. Phys..

[116] Wu X.D., Guo T., Liu L., Wang C., Zhang K., Liu H.Q., Wang F., Bai W.D., Zhang M.Y. (2017). MiR-23a targets RUNX2 and suppresses ginsenoside Rg1-induced angiogenesis in endothelial cells. Oncotarget.

[117] Cameron J.E., Fewell C., Yin Q., McBride J., Wang X., Lin Z., Flemington E.K. (2008). Epstein-Barr virus growth/latency III program alters cellular microRNA expression. Virology.

[118] Honegger A., Schilling D., Bastian S., Sponagel J., Kuryshev V., Sultmann H., Scheffner M., Hoppe-Seyler K., Hoppe-Seyler F. (2015). Dependence of intracellular and exosomal microRNAs on viral E6/E7 oncogene expression in HPV-positive tumor cells. PLoS Pathog..

[119] Saito A., Suzuki H.I., Horie M., Ohshima M., Morishita Y., Abiko Y., Nagase T. (2013). An integrated expression profiling reveals target genes of TGF-beta and TNF-alpha possibly mediated by microRNAs in lung cancer cells. PLoS ONE.

[120] Alarmo E.L., Havunen R., Hayrynen S., Penkki S., Ketolainen J., Nykter M., Kallioniemi A. (2016). Bone morphogenetic protein 4 regulates microRNA expression in breast cancer cell lines in diverse fashion. Genes Chromosom. Cancer.

[121] Wen Y.C., Lee W.J., Tan P., Yang S.F., Hsiao M., Lee L.M., Chien M.H. (2015). By inhibiting snail signaling and miR-23a-3p, osthole suppresses the EMT-mediated metastatic ability in prostate cancer. Oncotarget.

[122] Lu B., Sheng Y., Zhang J., Zheng Z., Ji L. (2016). The altered microRNA profile in andrographolide-induced inhibition of hepatoma tumor growth. Gene.

[123] Zhu M., Wang N., Tsao S.W., Yuen M.F., Feng Y., Wan T.S., Man K., Feng Y. (2011). Up-regulation of microRNAs, miR21 and miR23a in human liver cancer cells treated with Coptidis rhizoma aqueous extract. Exp. Ther. Med..

[124] Wang N., Zhu M., Wang X., Tan H.Y., Tsao S.W., Feng Y. (2014). Berberine-induced tumor suppressor p53 up-regulation gets involved in the regulatory network of MIR-23a in hepatocellular carcinoma. Biochim. Biophys. Acta.

[125] Zhang Y.C., Ye H., Zeng Z., Chin Y.E., Huang Y.N., Fu G.H. (2015). The NF-kappaB p65/miR-23a-27a-24 cluster is a target for leukemia treatment. Oncotarget.

[126] Khan A.U.H., Rathore M.G., Allende-Vega N., Vo D.N., Belkhala S., Orecchioni S., Talarico G., Bertolini F., Cartron G., Lecellier C.H. (2016). Human Leukemic Cells performing Oxidative Phosphorylation (OXPHOS) Generate an Antioxidant Response Independently of Reactive Oxygen species (ROS) Production. EBioMedicine.

[127] Tan X., Wang S., Zhu L., Wu C., Yin B., Zhao J., Yuan J., Qiang B., Peng X. (2012). cAMP response element-binding protein promotes gliomagenesis by modulating the expression of oncogenic microRNA-23a. Proc. Natl. Acad. Sci. USA.

[128] Takahashi H., Ishii H., Nishida N., Takemasa I., Mizushima T., Ikeda M., Yokobori T., Mimori K., Yamamoto H., Sekimoto M. (2011). Significance of Lgr5(+ve) cancer stem cells in the colon and rectum. Ann. Surg. Oncol..

[129] Bhushan L., Kandpal R.P. (2011). EphB6 receptor modulates micro RNA profile of breast carcinoma cells. PLoS ONE.

[130] He X.X., Kuang S.Z., Liao J.Z., Xu C.R., Chang Y., Wu Y.L., Gong J., Tian D.A., Guo A.Y., Lin J.S. (2015). The regulation of microRNA expression by DNA methylation in hepatocellular carcinoma. Mol. Biosyst..

[131] Wang Y., Zhang Z.X., Chen S., Qiu G.B., Xu Z.M., Fu W.N. (2016). Methylation Status of SP1 Sites within miR-23a-27a-24-2 Promoter Region Influences Laryngeal Cancer Cell Proliferation and Apoptosis. Biomed Res. Int..

[132] Mei Y., Si J., Wang Y., Huang Z., Zhu H., Feng S., Wu X., Wu L. (2017). Long Noncoding RNA GAS5 Suppresses Tumorigenesis by Inhibiting miR-23a Expression in Non-Small Cell Lung Cancer. Oncol. Res..

[133] Du Y., Weng X.D., Wang L., Liu X.H., Zhu H.C., Guo J., Ning J.Z., Xiao C.C. (2017). LncRNA XIST acts as a tumor suppressor in prostate cancer through sponging miR-23a to modulate RKIP expression. Oncotarget.

[134] Wang Z. (2011). The guideline of the design and validation of MiRNA mimics. Methods Mol. Biol..

[135] Ying S.Y., Chang D.C., Miller J.D., Lin S.L. (2006). The microRNA: Overview of the RNA gene that modulates gene functions. Methods Mol. Biol..

[136] Ortega M.M., Bouamar H. (2017). Guidelines on Designing MicroRNA Sponges: From Construction to Stable Cell Line. Methods Mol. Biol..

